# The Vascular Factor Plays the Main Role in the Cause of Pain in Men with Chronic Prostatitis and Chronic Pelvic Pain Syndrome: The Results of Clinical Trial on Thermobalancing Therapy

**DOI:** 10.3390/diseases5040025

**Published:** 2017-11-08

**Authors:** Simon Allen

**Affiliations:** Fine Treatment, 29 Rewley Road, Oxford OX1 2RA, UK; info@finetreatment.com; Tel.: +44-7958-878300; Fax: +44-186-572-8255

**Keywords:** chronic prostatitis, chronic pelvic pain syndrome, cause of pain, prostate treatment, thermobalancing therapy

## Abstract

Chronic pain in patients with chronic prostatitis/chronic pelvic pain syndrome (CP/CPPS), NIH category III is difficult to treat without understanding its cause. The main symptom of chronic prostatitis is pain. In this study, we would like to explain the origin of pain in men with CP/CPPS and its therapy. Forty-five patients with CP/CPPS have received thermobalancing therapy (TT) enabled by Dr Allen’s therapeutic device (DATD) for six months as mono-therapy. The control group comprised 45 men with CP/CPPS did not receive TT. Before and after six months the National Institute of Health Chronic Prostatitis Symptom Index (NIH-CPSI) scores, prostatic volume (PV) by ultrasound measurement and uroflowmetry (Q_max_) were compared between the groups. Baseline characteristics have shown no difference. After TT, significant improvements in pain score (*p* < 0.001), quality of life index (QoL) (*p* < 0.001), decrease of PV (*p* < 0.001), and increase Q_max_ (*p* < 0.001) were determined. There were not noteworthy changes in the control group. Chronic pain due to CP/CPPS happens as a consequence and challenges at the capillary level, namely pathological capillary activity. In response to initial triggers—such as inflammation, cold, psychological and other factors—constriction and spontaneous expansion of capillaries follows, creating a continuous secondary trigger—i.e., the micro-focus of hypothermia—which in turn provokes expansion of capillaries. The additional tissue due to vascular changes into the prostate increases pressure on nociceptors causing pain. TT relieves chronic pelvic pain by eliminating the lasting focus of hypothermia in the affected prostate tissue.

## 1. Introduction

Chronic inflammation predicts risk progression of pain in men who have identified chronic prostatitis/chronic pelvic pain syndrome (CP/CPPS) symptoms [1]. However, no singular cause of CP/CPPS has been recognized, therefore it is most likely a syndrome with multifactorial causes. Thus appropriate treatment for chronic pelvic pain is a multimodal approach based on phenotyping including alpha-blockers, antibiotics, anti-inflammatory medication, hormonal therapy, phytotherapy, antispasmotics, and non-drug-related strategies, such as psychotherapy and attempts to improve relaxation of the pelvic floor [2,3].

Absence of effective therapy for CP/CPPS and understanding of its etiology led to a search for the mechanisms of the pain in psychological status of males [4]. Increased pain is associated with worse quality of life (QOL) and greater overall symptom presentation in men with CP/CPPS, hence different illness-focused coping strategies were suggested, including catastrophizing pain-contingent rest and cognitive-behavioural self-management interventions [5,6].

It was reported that cold is one of the factors that can trigger a process resulting in CP/CPPS and furthermore is responsible for symptom aggravation [7]. At the same time heat can decrease pain that can occur via vasodilatation, improved dilatation of muscles or stimulation of sensory receptors in the skin [8]. Transcutaneous electrical nerve stimulation (TENS) is a noninvasive intervention used in rehabilitation to modulate pain but skin impedance was not a factor in TENS effect [9]. So there have been attempts to study the effect of temperature changes on the occurrence and course of the CP/CPPS symptoms, including pain. However, only recent research on thermobalancing therapy (TT) has shown that a source of energy with the body temperature directed to the affected prostate gland eliminates the focus of hypothermia in relieving pain [10].

It has been explained that in response to irritations—i.e., initial triggers in development of chronic prostatitis—two functional physiological properties of small blood vessels, named by physiologists: constriction of capillaries and spontaneous expansion of capillaries, are activated. Firstly, capillary constriction creates micro-focus of hypothermia, which later becomes a continuous irritant. Secondly, in response to irritation (i.e., a trigger-initiator and later focus hypothermia) blood flow increases through the spontaneous expansion of the capillary net locally [11,12]. Thus, pathological capillaries activity is established. The formation of new capillaries is essentially the growth of the excess tissue that leads to an increased pressure inside the prostate tissue, which in turn provokes new constrictions of the capillary net. It makes prostatic disease and pain chronic. For the treatment of chronic internal diseases, TT enabled by Dr. Allen’s therapeutic device (DATD) was created [13]. The first clinical study on TT in men with lower urinary tract symptoms (LUTS) due to benign prostatic hyperplasia (BPH) has shown effectiveness of new therapy [14,15]. In this article, we would like to discuss that chronic pain, like other symptoms and characteristics of CP/CPPS, may appear and progress because of changes at the capillary level in the prostate tissue in the first place.

## 2. Methods and Results

### 2.1. Study Design

Comparing 45 men with CP/CPPS who received treatment with DATD for a six-month period with the control group, comprised of 45 men who did not receive the therapy, studied the effectiveness of thermobalancing therapy. The clinical controlled observational study was used as most men with CP/CPPS have serious mental problems that make it practically impossible to hide the essence of the device and to use plastic or something else will not help [16]. Secondly, the goal of our investigation was to determine if long term application would provide pain relief but we did not determine what material should be used for the device.

### 2.2. Participants

Over two years, a total 45 men (age < 55 years) with CP/CPPS were selected for the clinical trial at The Department of Urology of the Medical University. The patient selection was achieved in a multidisciplinary manner in conjunction with urologist and written informed consent for participation in the study was obtained. Two investigated groups were statistically homogeneous. Inclusion criteria were men with chronic prostatitis and pain evaluation score ≥1, according to the NIH-Chronic Prostatitis Symptom Index. Exclusion criteria were acute prostatitis; urethral stricture, neurogenic bladder, and co-morbidities, such as impaired renal function and diabetes mellitus. We included patients with serum prostatic specific antigen (PSA) higher than 4 ng/mL in which, after the prostate biopsy, there was no evidence of cancer. Medical treatments at the time of enrolment were not taken into consideration.

### 2.3. Evaluation

The effectiveness of thermobalancing therapy was studied by comparing men with CP/CPPS who received treatment with therapeutic device as mono-therapy with the control group, who received no treatment. The baseline evaluations included complete physical examination, medical history, DRE, serum biochemistry, PSA measurements, electrolytes, urine, and renal function tests. Evaluations were made at baseline and six months after the treatment. At the baseline assessment, patients were evaluated for NIH-Chronic Prostatitis Symptom Index (NIH-CPSI), pain, and QoL scores, PV (mL), and uroflowmetry, maximum urinary flow rate (Q_max_), mL/s. Dynamics of the symptoms and indicators in each group were assessed at the beginning and at the end of the treatment period by using NIH-CPSI. Ultrasound was used to determine the volume of the prostate gland (US-9000E2 ultrasound scanner, Rising Medical Equipment Co. Ltd., Beijing, China). The standard ellipsoid formula, length × width × height × 0.52, was used to determine PV.

### 2.4. Statistical Analysis

The independent-samples *t*-test and paired-samples *t*-test are suitable only for interval and ratio data, so the Wilcoxon signed-rank test was employed. A value of *p* < 0.05 was considered significant. Statistical analyses were carried out using SPSS v22 (IBM, Armonk, New York, NY, USA).

### 2.5. DATD

**Figure 1:**
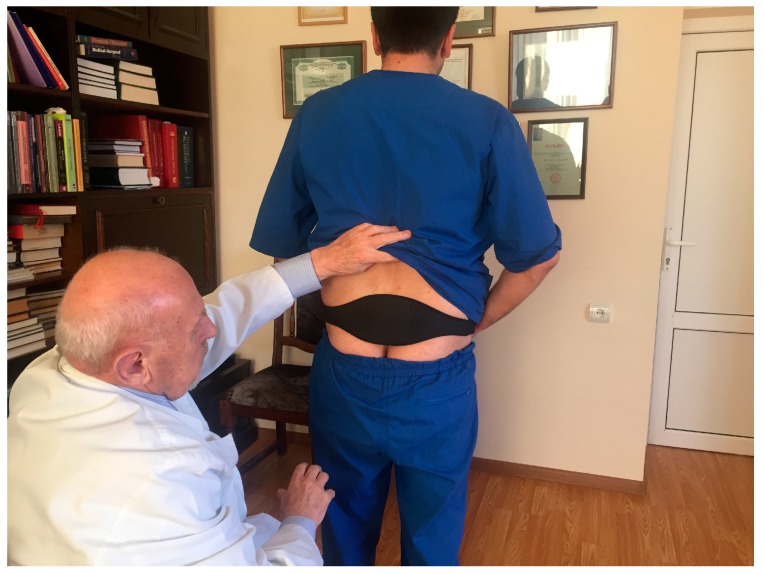
The therapeutic device is placed on the patient’s body.

Men in the treatment group after the screening were given physiotherapeutic device. This physiotherapeutic device provides a method of treating an affected prostate by the application of a special mixture of waxes (thermoelement) topically to the skin in its projection. This thermoelement is able to accumulate the emitted body heat, and thus turns into a source of energy itself. Thus, DATD provides TT. It applies the thermoelement tightly to the coccyx area, so it overcomes the skin barrier, spreading energy toward the prostate. DATD is an elastic neoprene belt that keeps a thermoelement in the projection of prostate for a prolonged period of time. The thermoelement allows body heat accumulation and acts as the heat source for the prostate. The neoprene belt keeps the thermoelement tightly applied to the skin and does not allow heat dissipation (Figure 1).

The production of the therapeutic device started in 2010 in England. In April 2010, the device was registered at the medicines and healthcare products regulatory agency (MHRA) as class 1 medical device. Class I Medical Device without a measuring function and supplied in non-sterile condition does not require the involvement of a Notified Body. In accordance with the ‘Regulation of medical devices outside the European Union’, low-risk products may only require a supplier’s declaration of conformity (SDOC), where the manufacturer is responsible for ensuring that the product complies with the relevant requirement and then produces a written self-declaration statement [17].

## 3. Results

### 3.1. Prostate Volume and Pain Score

**Figure 2:**
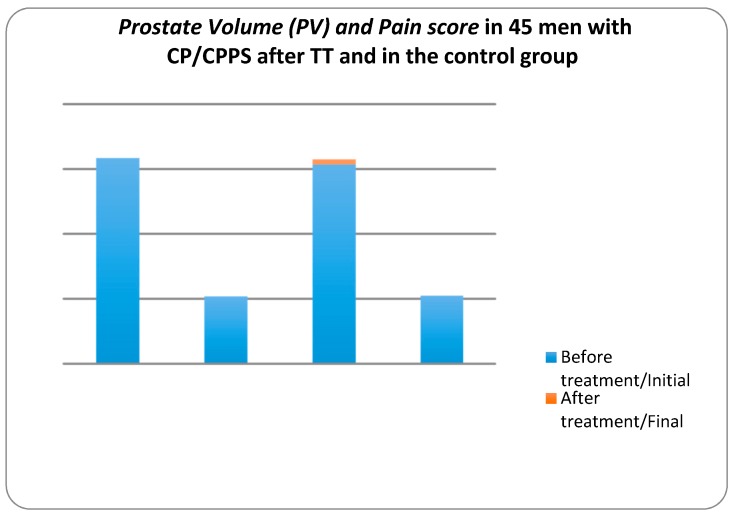
Dynamics of Pain scores and Prostate Volume (PV) mL in 45 men with CP/CPPS after TT and in the control group measured by the National Institute of Health-Chronic Prostatitis Symptom Index (NIH-CPSI) and ultrasound.

Figure 2 shows the changes in PV mL in CP/CPPS patients. In the control group, the mean prostate volume increased from 30.77 ± 6436 to 31.58 ± 7.138 mL at the end of the study period, whereas in the treatment group the mean PV decreased from 31.75 ± 7.009 to 27.07 ± 4.522 mL. For the treatment group, the z value was −5.392 at the significance level *p* value < 0.001. These data indicated that the therapeutic device reduced the prostate volume significantly, whereas in the control group the prostate volume increased.

Figure 2 also shows the changes in pain score in CP/CPPS patients at the beginning and at the end of the study. In the control group, the mean of pain score decreased from 10.49 to 9.71 at the end of the study period, whereas in the treatment group the mean of pain score decreased from 10.38 to 3.58. In the treatment group, the z value is −5.725 at the significance level *p* value < 0.001. These data suggest that the pain score decreased in groups. However, pain in the ‘treatment’ group decreased considerably while in the ‘no treatment’ group it only decreased slightly.

### 3.2. Quality of Life and Maximum Urinary Flow Rate

**Figure 3:**
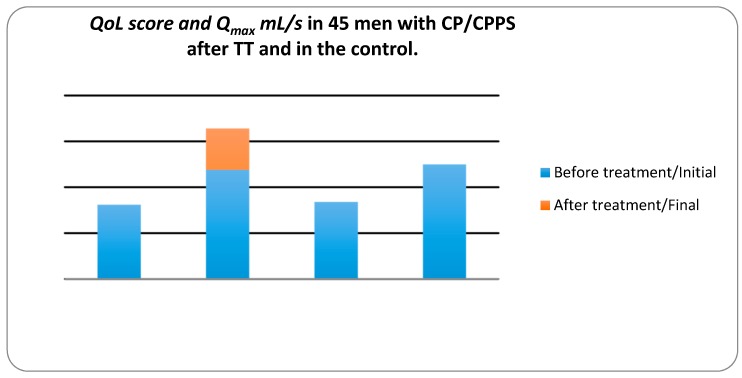
Quality of life (QoL) score and maximum urinary flow rate (Q_max_) mL/s in 45 men with CP/CPPS after TT and in the control group measured by the National Institute of Health-Chronic Prostatitis Symptom Index (NIH-CPSI) and uroflowmetry.

We assessed the QoL according to NIH-CPSI (see Figure 3). In the control group, the mean QoL decreased slightly from 8.47 to 8.33, whereas in the treatment group the mean QoL decreased from 8.11 to 2.98. For the treatment group, the z value was −5.661 at the significance level *p* value < 0.001. These results indicated that the treatment with therapeutic device decreased the QoL significantly while in the control group it decreased slightly.

In the treatment group Q_max_ mL/s, increased from 11.93 ± 4.344 to 16.45 ± 3.503 mL/s, the z value was −5.249 at a significance level *p* < 0.001. In the control group, mean ± SD Q_max_ decreased from 12.59 ± 3.572 to 12.20 ± 2.543 mL/s. These results suggest that TT increases Q_max_ significantly in CP/CPPS patients whereas, in the control group no changes in Q_max_.

## 4. Discussions

### 4.1. Outcomes

As we can see on Figure 1 it is easy to wear DATD. The device is tightly applied to the coccyx area and does not impede movements. Figure 2 shows that continuous use of TT has reduced PV and pain. We believe that these two parameters are connected. According to Dr Allen, the origin of chronic internal diseases lies at the capillary level, as pathological capillaries activity increases pressure in the prostate tissue. Indeed, this pressure leads to chronic pressure on nociceptors causing pain [18]. Mehik and colleagues have reported that increased intraprostatic pressure in men with CP/CPPS plays the main role in pelvic pain and other symptoms [19]. Reducing the tissue stress may reduce the size of the prostate, and it is the key for pain relief. Figure 3. demonstrates improvement of QoL and increase Q_max_, which are additional parameters for the assessment of TT, confirming connection between pain, QoL, and changes in prostate.

### 4.2. Quality of Life

It is usual that men with CP/CPPS report significant deterioration in QoL [20,21,22]. No improvement after different medical interventions and CP/CPPS progression may lead to negative endpoints associated with pain: the deterioration of quality of life and even physical disability [23,24]. The reduction of the PV after use of DATD led to a significant decrease of pain and urinary symptoms with an improvement of QoL in patients with an enlarged prostate and CP/CPPS [25]. These facts indicate that it is prostate lesions that are the cause of pain in men with CP/CPPS and impair their quality of life.

### 4.3. Prostate Volume

Prostate size does not appear to be a significant risk factor for CP/CPPS, therefore it has not been recommended for the evaluation of patients with this disease [26]. However, researchers have noted that some prostate enlargement in men with CP/CPPS. For instance, at MR spectroscopic imaging pathologically confirmed chronic prostatitis may demonstrate metabolic abnormality, and it was practically not possible to find differentiation of chronic prostatitis from low-grade prostate cancer [27,28,29]. Therefore, in order to reduce the size of prostate gland different drugs and even minimally invasive surgical options were used not long ago for men with CP/CPPS [30]. Our findings confirmed that TT stimulates the restoration processes in prostate by influencing the etiology of CP/CPPS, therefore can be recommended as an independent and traditional treatment option for these patients [31].

### 4.4. Etiology

Many different mechanisms of pathogenesis of CP/CPPS symptoms have been discussed, such as immunological, neurological, endocrine, and psychological factors [32]. Cho IR with colleagues in 2000 determined abnormal prostate blood flow, increased blood flow to the prostatic capsule and diffuse flow throughout the prostatic parenchyma, in men with CP/CPPS [33]. Later increased arterial stiffness, vascular endothelial dysfunction, and chronic ischemia in the prostate tissue was reported [34,35]. However, most researchers did not pay attention to changes in blood flow in men with CP/CPPS. The use of DADT by keeping the source of body temperature in the projection of the prostate gland for a prolonged period has deactivated the micro-focus of hypothermia and ischemia, thereby relieving the pressure in the prostate tissue and pain [36]. We also investigated the influence TT on BPH and guided that improvement of blood circulation at the capillary level plays a crucial role in its cause [37].

### 4.5. Prevalence

Pelvic pain and decreased QoL are common complaints in middle-aged men. It was found that CP/CPPS associates with previously diagnosed anxiety disorder and a number of men with CP/CPPS as symptoms increase [38]. CP/CPPS is a condition that causes severe symptoms and worsens QoL in 8.0% of to 14% of men [39,40]. CP/CPPS has a significant impact on sexual dysfunction—including erectile dysfunction, ejaculatory pain, and premature ejaculation [41]—and a significant negative effect on sperm concentration, sperm progressive motility, and normal sperm morphology, thus impairing male fertility [42]. Therefore, in men with CP/CPPS, the main symptom of which is pain, may suffer from catastrophizing, stress, and co-morbid psychiatric disorders such as depression, anxiety, and others.

## 5. Conclusions

Pain is the dominating symptom in men with CP/CPPS and is associated with worse QoL with impact on physical and mental factors. The vascular changes with the focus of hypothermia in affected prostate tissue play the main role in the cause the pain. TT eliminates this hypothermia by maintaining a temperature that does not exceed body temperature and treats prostate tissue, relieving pressure on stress nociceptors, which results in relief of pelvic pain. Thus, the cause of pain is the pathological activity of the capillaries, which is initiated by various initial triggers and a secondary trigger which is the focus of hypothermia in the prostate tissue, which creates pressure inside the prostate, causing pelvic pain.
